# Progress in Surgery

**Published:** 1899-10-07

**Authors:** 


### Progress in Surgery.

### GENITO-URINARY SURGERY.

**Renal Calculi.**—The detection of renal calculi by the X-rays has been, and is still, a matter of considerable uncertainty. As Abbe says, doubt about the existence of a renal calculus can only be settled by exploratory nephrotomy or by radiography.[1] The former is a fairly exact method, and if no stone be found there is usually some other cause for the symptoms. Thus, Henry Morris records 44 cases in which no stone was found, although many of the patients were relieved. Abbe gives a total of 27 recorded cases in which skiagraphy has been useful, and adds two cases of his own. In one case the calculus could not for long be felt even with the finger in the renal pelvis, but the skiagram was so convincing that a continued search revealed a rough calculus in a pocket. Abbe finds short exposures to be more successful than long ones. C. Mansell Moullin has considered the radiography of renal calculi.[2] For some purposes he regards the fluorescent screen as of more value than the skiagram.

**Renal Tuberculosis.**—An interesting discussion on nephrotomy *versus* nephrectomy for tuberculosis of the kidney was started by Dr. Kammerer.[3] Most of the speakers were in favour of primary nephrectomy for tuberculous kidney. Many thought the ureter was well able to take care of itself and need not be excised. Some thought a preliminary nephrotomy often an exceedingly good measure, preparing the patient for the severer operation of nephrectomy later. According to Israel, renal tuberculosis appears to be primary in three-fourths of the cases, and is bilateral in only one-eighth of the cases, although some find the proportion to be nearly one-half. Tuberculosis of the bladder co-exists in nearly 50 per cent. of cases. Israel does not regard nephrectomy as admissible in the majority of cases, more particularly when the bladder is also involved. Dr. Alexander Johnson records nine cases of tuberculosis of the kidney. In the majority the symptoms were referred to the bladder, and in two cases the symptoms simulated those of calculus precisely.[4] Eight cases were treated by primary nephrectomy, of which one died from the operation, one died on the tenth day, and one on the forty-seventh day, leaving five cases to survive for longer or shorter periods.

**The Ureter.**—No surgeon has given so much attention to the surgery of the ureter in this country as Mr. Henry Morris. He recalls some 26 out of 47 cases tabulated in 1876, in which, in the present day, the kidneys might have been preserved. Morris has operated on five such cases in the last few years.[5] He gives an elaborate *résumé* of published cases and of the various operations, which it is impossible to detail here. The methods of exposing the renal pelvis and ureter have been elsewhere described.[6] The main portion of the ureter may be reached by a transperitoneal or extraperitoneal operation, and the pelvic portion of the ureter by at least seven routes. When in doubt as to which ureter is involved the median transperitoneal route is preferable. When it is a question as to which part of one ureter is involved, then a lateral transperitoneal operation may be done. But transperitoneal operations are imperfect and incomplete. The extraperitoneal method enables one to explore the whole length of the ureter. The pelvic portion is exposed in women by either the vesical or vaginal route.

**Enlarged Prostate.**—Parker Syms has a useful paper on prostatectomy.[7] There are at least seven ways of performing this operation. Suprapubic operations have the disadvantage of leaving an ulcer in the base of the bladder, and suitable perineal prostatectomy does not provide the much-needed drainage. The author regards perineal prostatectomy, combined with suprapubic cystotomy and perineal drainage, as the best operation. The floor of the bladder is not wounded, the perineum is cut in the middle line by a single incision, time is saved, and the hæmorrhage is minimised. The rectum being empty, the bladder is distended as usual, and a suprapubic opening made so as to admit two fingers for manipulation. The entire prostate is shelled out from its capsule through the perineal wound. The lateral lobes are first removed, and subsequently any median growth. A drainage tube is inserted into the bladder through the membranous urethra, and another is placed in the suprapubic opening, the latter being removed on the fourth day, the bladder having meanwhile received daily irrigations. The author's experience leads him to regard suprapubic drainage as a very undesirable procedure in old men. Not only is the drainage uphill, but there is a very great tendency to infection of the loose areolar tissue in the prevesical space. When successful the results of prostatectomy are very good; indeed, they are better than those of any other measure. The mortality, however, is high, although this is probably due to the fact that the operation is performed in the worst cases, and in those left until a late stage in the disease is reached. Alexander has twice performed the operation in thin subjects by enucleation from the perineum, without any wound above the pubes, by pressing upon the lower abdominal wall instead.

We have previously pointed out the revival of Bottini's operation in America. Guitéras has since published a dozen cases of prostatic enlargement benefited by the operation.[8] At first sceptical as to results, the author has been more than pleased with the progress of the cases. He holds that operation should be done as soon as bladder-strain begins, and we should not wait for complete retention and cystitis. He calls attention to the danger of drawing off all the residual urine at the first attempt, and to the danger which may result from cystoscopy. He prefers general anæsthesia under nitrous oxide gas. Chloroform is dangerous, ether is bad for the kidneys, and cocaine has some dangers and is inadequate. According to his experience anterior and posterior cuts are better than lateral ones in their results, and the incisions should vary from one to one and three-quarter inches in length. The effect of the cauterisation is to groove the prostate and to cut off some of the blood-supply, and thus induce atrophy. Sloughs are subsequently passed per urethram.

It is important that a patient with complete retention should never be operated upon until accustomed to catheter-life. The burning feeling and tenesmus are apt to become inconvenient afterwards. Guitéras, like Willy Meyer, regards this as the best operation for prostatic hypertrophy.

More discrimination is urged by Reginald Harrison in the selection of cases for the various operations, so that the operation may correspond with the type of enlargement.[9] Harrison gives three types: Firstly, the engorged soft prostate; secondly, the fibrotic and pedunculated prostate; and, thirdly, the adenomatous prostate. It is for the first condition that vasectomy appears to be indicated. It particularly reduces the tendency to vesical irritability.

**The Bladder.**—Catheterisation of the ureters seems to have established itself as a most useful procedure. It has been extended by Casper to the local treatment of the kidney in cases of pyelitis, so called. However, there seems to have been considerable discussion at the Berlin Medical Society as to the utility of the process, some taking one side snd some the other. Israel strongly opposed it as giving no reliable information when nephrectomy is contemplated.[10] Kreps, on the other hand, emphasises its value, particularly in distinguishing between cystitis and pyelitis, and in ascertaining which of two kidneys is the diseased one, and whether the other be healthy.[11]

The value of the cystoscope in genito-urinary surgery is becoming recognised. Thelwall Thomas has recorded four cases in which the diagnosis and nature of the tumours was made by means of the cystoscope.[12]

An unusually large collection of cases of encysted vesical calculi has been described by Mr. Bruce Clarke, the majority being under his own care.[13] He calls attention to the fact that encysted calculi do not produce the pain and hæmaturia which characterise free calculi. Of the six cases first described no fewer than three had stones with fragments of bone for their nuclei, which had probably come from neighbouring diseased structures. Some of his cases throw doubt on the view that sacculi are herniæ of the mucous membrane, for he has found muscular tissue in their walls. After removal of the calculi some sacculi tend to cicatrise spontaneously; others may require drainage by the perineum or rectum.

**Reinfection in Syphilis.**—Contrary to previous ideas the possibility of reinfection seems to be established. Casaline has recorded a case of a prostitute who had a second attack of syphilis, with a primary sore, before the manifestation of the first attack had passed away.[14] Several other cases have been referred to of late. Cases recorded formerly are not reliable, owing to the confusion between soft sores and chancres.[15]

[1] Annals of Surgery, Aug., 1899. [2] Lancet, May 27, 1899. [3] Annals of Surgery, June, 1899, p. 753. [4] Annals of Surgery, Feb., 1899, p. 226. [5] Lancet, July 1, 1899. [6] Clin. Jour., June 28, 1899. [7] Annals of Surgery, May, 1899. [8] New York Med. Rec., July 29, 1899. [9] Lancet, Aug. 5, 1899. [10] Lancet, Jan. 28, 1899. [11] Med. Rec., Mar. 18, 1899. [12] Brit. Med. Jour., June 3, 1899. [13] Brit. Med. Jour., May 13, 1899. [14] Epit. Brit. Med. Jour., April 15, 1899. [15] Brit. Med. Jour., April 15, 1899.

